# Evaluating passive immunity in piglets from sows vaccinated with a PEDV S protein subunit vaccine

**DOI:** 10.3389/fcimb.2024.1498610

**Published:** 2025-01-31

**Authors:** Jiajia Liu, Guangli Hu, Shengjin Liu, Guangcai Ren, Liguo Gao, Zhiqing Zhao, Rui Geng, Dingli Wang, Xiao Shen, Feng Chen, Hanqin Shen

**Affiliations:** ^1^ Guangdong Enterprise Key Laboratory for Animal Health and Environmental Control, Wen’s Foodstuff Group Co. Ltd, Yunfu, China; ^2^ Yunfu Branch, Guangdong Laboratory for Lingnan Modern Agriculture, Yunfu, China; ^3^ State Key Laboratory of Biocontrol, School of Life Sciences, Sun Yat-sen University, Guangzhou, China; ^4^ College of Animal Science, South China Agricultural University, Guangzhou, China; ^5^ Canton Biologics Co., Ltd, Guangzhou, China

**Keywords:** porcine epidemic diarrhea virus, PEDV, spike protein, immunogenicity, subunit vaccine

## Abstract

Porcine epidemic diarrhea virus (PEDV) is a highly contagious virus that causes severe diarrhea and high mortality in neonatal piglets. Current control measures, such as inactivated and live-attenuated vaccines, have limitations in providing complete protection. In this study, we evaluate the immunogenicity and protective efficacy of a PEDV S protein subunit vaccine compared to a traditional inactivated vaccine. Piglets and Sows were immunized with either the subunit vaccine or an inactivated vaccine, and serum samples were collected to assess IgG and neutralizing antibody levels. Results demonstrated that the S protein subunit vaccine induced significantly higher IgG and neutralizing antibody levels in both piglets and sows compared to the inactivated vaccine. Piglets born to immunized sows were challenged with a virulent PEDV strain. Piglets from the subunit vaccine group exhibited lower viral shedding, reduced clinical symptoms, and minimal intestinal lesions. These findings suggest that the PEDV S protein subunit vaccine provides enhanced immunity and protection against PEDV, making it a promising candidate for preventing PEDV infections in swine.

## 1 Introduction

Coronaviruses are a diverse group of enveloped, single-stranded RNA viruses known for their ability to infect a broad range of hosts, including humans (Wang et al., 2019). Some coronaviruses cause high pathogenicity in humans, including severe acute respiratory syndrome coronavirus (SARS-CoV), Middle East respiratory syndrome coronavirus (MERS-CoV), and the most recent and impactful, SARS-CoV-2, responsible for the COVID-19 pandemic (Zhu et al., 2020). Coronaviruses also cause severe disease in pigs, such as porcine epidemic diarrhea virus (PEDV), transmissible gastroenteritis virus (TGEV) and porcine deltacoronaviruses (PDCoV) (Kong et al., 2022). PEDV is an enveloped, positive-sense single-stranded RNA virus from the *Alphacoronavirus* genus within the *Coronaviridae* family (Madson et al., 2014; Pensaert and de Bouck, 1978), which causes severe diarrhea, vomiting, dehydration, and high mortality, especially in neonatal piglets (Sun et al., 2012). Since its emergence, PEDV has caused substantial economic losses in the swine industry worldwide (Chen et al., 2014; Trujillo-Ortega et al., 2016; Yang et al., 2018; Zhang et al., 2023). The virus primarily infects the small intestine, leading to villous atrophy and impaired nutrient absorption, which results in malnutrition and dehydration in infected piglets (Song et al., 2024). Despite the availability of vaccines, current options, including inactivated and live-attenuated vaccines, have limitations in providing complete protection, particularly in severe outbreaks.

The genome of PEDV, approximately 28 kilobases in length, encodes several structural proteins, including the spike (S), envelope (E), membrane (M), and nucleocapsid (N) proteins (Wang et al., 2019). Among these, the S protein plays a crucial role in viral attachment and entry into host cells and is a key antigen for inducing neutralizing antibodies, making it an ideal target for vaccine development (Song et al., 2024). Recent research has focused on the development of subunit vaccines utilizing the S protein to enhance immunogenicity while minimizing the risks associated with live virus vaccines (Zhao et al., 2024). Subunit vaccines offer significant safety advantages by using specific viral components rather than the whole virus, thus reducing the risk of adverse effects.

In precent study, we evaluated the immunogenicity and protective efficacy of a PEDV S protein subunit vaccine compared to a traditional inactivated vaccine. By monitoring clinical symptoms, viral shedding, and intestinal lesions in piglets challenged with a virulent PEDV strain, this study seeks to determine whether the S protein subunit vaccine can provide superior protection. Additionally, the transfer of maternal antibodies to piglets through colostrum and its impact on piglet immunity will be assessed to evaluate the vaccine’s effectiveness in providing passive protection against PEDV.

## 2 Materials and methods

### 2.1 Cells and virus

Chinese hamster ovary (CHO) K1 cells were purchased from ECACC and maintained in CHO-rise^®^ (Biologics, China). Vero cells were cultured in DMEM supplemented with 10% fetal bovine serum (Gibco, USA) at 37°C with 5% CO2. PEDV hddz strain (GenBank accession number: PQ316088) was passaged in vero cells with DMEM supplemented with 7 µg/mL trypsin.

### 2.2 Expression and purification of PEDV S protein

The PEDV (strain: dndz) spike ectodomain (S-ECD) was fused with a T4 foldon to stabilize its trimeric conformation, expressed in CHO cells, and purified for use as vaccine antigens. Briefly, the DNA fragment encoding the S-ECD (amino acids 1 to 1325) of PEDV, with the T4 foldon fused at the C-terminus, was synthesized and cloned into the pMGT expression vector, which includes a glutamine synthetase (GS) selectable marker. The plasmid was then transfected into CHO cells, followed by selection with methionine sulfoximine (MSX) to generate stable cell pools for consistent spike protein production. For protein purification, the supernatant collected from the cell culture was desalted using 20 mM phosphate-buffered saline (PBS), pH 7.5, and applied to an anion-exchange chromatography column. The protein was eluted using a gradient of 0 to 0.5 M NaCl, and the fractions containing the spike protein were pooled. The pooled protein was then ultrafiltered into 20 mM phosphate buffer (PB), 150 mM NaCl, pH 7.5, and further purified using size exclusion chromatography (SEC) to remove residual host cell proteins. The purified PEDV-S protein was analyzed by SEC-HPLC and transmission electron microscopy (TEM).

### 2.3 Preparation of recombinant subunit vaccine

To prepare the S protein subunit vaccine, the purified recombinant S protein was mixed in a 1:1 (w/w) ratio with MONTANIDE™ ISA 201 VG adjuvant (SEPPIC, France) and emulsified according to the manufacturer’s instructions. The final vaccine contained 25 μg/mL of S protein. For virus inactivation, the PEDV dndz viral suspension (titer of 10^7.5^ TCID_50_/mL) was treated with methyl aldehyde at a final concentration of 0.1% and incubated at 37°C for 24 h. Virus inactivation was confirmed by inoculating the treated samples into Vero cells and serially passaging them for two generations. The inactivated PEDV was then mixed with ISA 201 VG adjuvant, and the final inactivated PEDV vaccines were emulsified and prepared for use.

### 2.4 Assessment of immunogenicity of recombinant protein in piglets

The experimental protocol was previously approved by the South China Agricultural University Experimental Animal Ethics Committee (approval ID: SYXK-2019-0136) and was performed in accordance with relevant guidelines and regulations.

Fifteen conventional 21-day-old piglets were obtained from a pig farm that was negative for PEDV, TGEV, PoRV, and PDCoV fecal swabs by RT-PCR testing. All the piglets were also confirmed PEDV negative by serum antibody enzyme-linked immunosorbent assay (ELISA). All the experimental animals were housed in temperature-controlled isolation rooms and had free access to feed. The piglets were randomly divided into Three groups, with five piglets in each group. Group A was immunized with 50 μg (2 mL) 201 adjuvanted S protein vaccine, Group B was immunized with 201 adjuvanted PEDV hddz strain inactivated vaccine, and Group C was served as mock control group. The piglets were boost immunized 2 weeks post first immunization. The sera were collected every week for IgG antibody and neutralizing antibody testing.

### 2.5 Assessment the passive protection of recombinant protein in piglets

**Figure 1:**
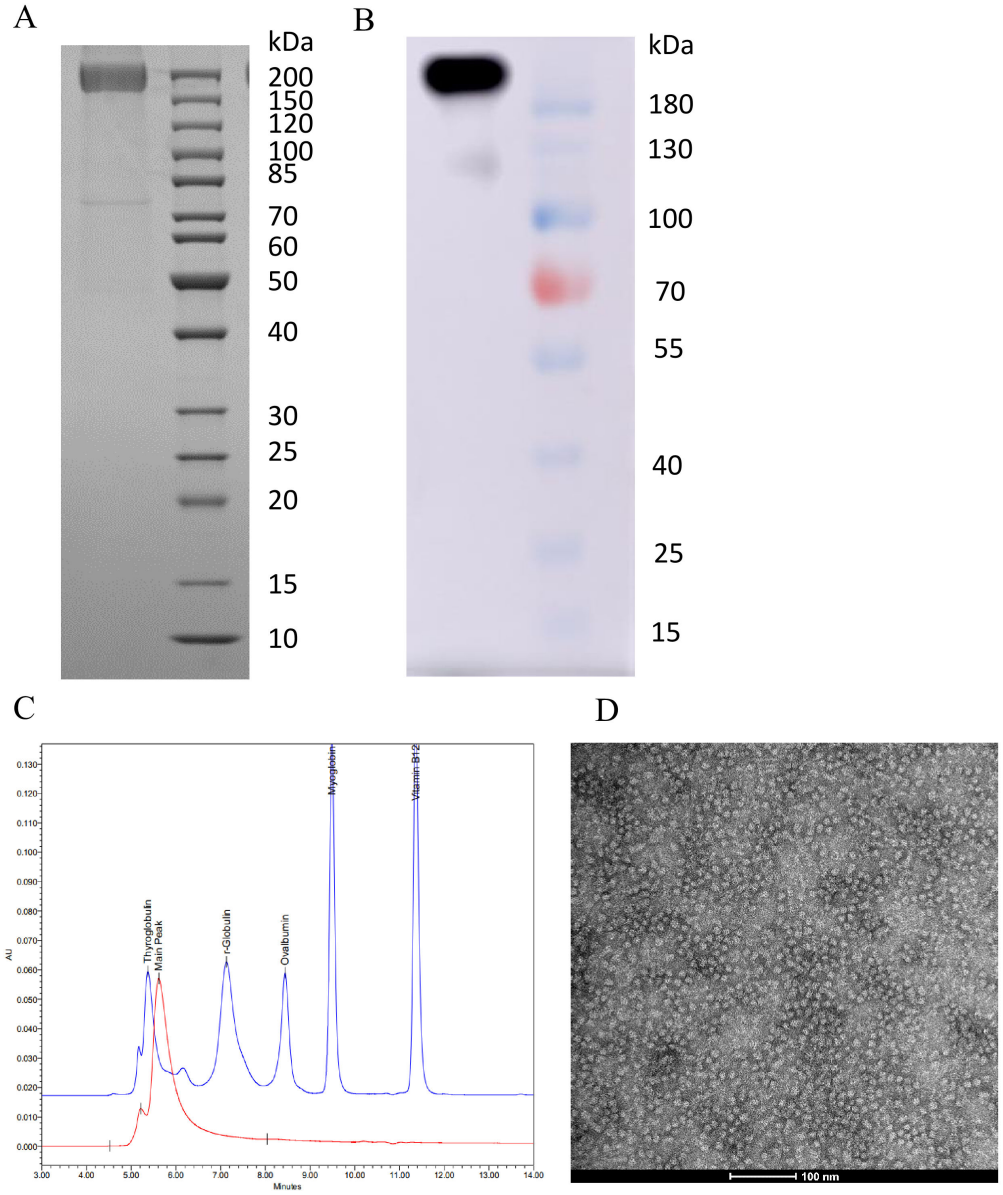
Expression of PEDV S protein in CHO. **(A)** SDS-PAGE analysis of purified S protein. **(B)** Western blot analysis of purified S protein. **(C)** SEC-HPLC analysis of purified S protein (red line). **(D)** TEM analysis of the purified S protein.

To evaluate the protective efficacy of the PEDV S protein subunit vaccine, 9 conventional sows were obtained from a pig farm that was negative for PEDV, TGEV, PoRV, and PDCoV fecal swabs by RT-PCR testing. All the sows were confirmed PEDV negative by antibody ELISA (serum). The 9 sows were randomly divided into three groups, each consisting of 3 sows. The immunization schedule was shown in **Figure 1A**. Three sows were immunized with the PEDV S protein subunit vaccine (50 μg/2 mL) at 5 weeks and 3 weeks before farrowing. Another group of three sows received 2 mL of inactivated PEDV vaccine at the same time points. The remaining three sows were given 2 mL of PBS in ISA 201 VG adjuvant as a negative control. Serum samples were collected from the sows at various time points post-immunization, and colostrum samples were collected after farrowing for antibody detection. After 5 days of suckling, 8 piglets were randomly selected from each group. These piglets were orally challenged with the PEDV hddz strain at a dose of 1×10^6.0^ TCID_50_ per piglet. Five piglets from each group had rectal swabs collected daily for viral detection, and fecal consistency was scored to monitor clinical signs. The remaining 3 piglets from each group were necropsied 3 days post-challenge to collect intestinal tissues and contents. These samples were subjected to histopathological evaluation for lesions and quantitative detection of PEDV RNA to assess viral load.

### 2.6 Indirect enzyme-linked immunosorbent assay

PEDV S protein specific IgG antibody in piglet or sow serum was detected by ELISA using PEDV IgA Antibody Test Kit (DHN, China) according to the manufacturer. PEDV S protein specific IgA antibody in sow serum or colostrum was detected using PEDV IgA Antibody Test Kit (DHN, China). In short, Serum or colostrum samples were diluted at a specific ratio using sample dilution buffer. Then, 100 μL of the diluted samples were added to the reaction plates and incubated at 37°C for 1 h. After incubation, the wells were washed three times, followed by the addition of 100 μL of enzyme-labeled secondary antibody. The plates were incubated again at 37°C for 1 h. After three times washes, a substrate solution was added and incubated at 37°C for 10 minutes. The absorbance was then measured, and the sample’s S/P ratio was calculated for analysis.

### 2.7 Viral real-time quantitative PCR

The Viral real-time quantitative PCR (RT-qPCR) was performed as previously described (Wu et al., 2019). Total RNAs were extracted using Rneasy Mini Kit (Qiagen), and reverse transcription was conducted using the Super Script III First-Strand Synthesis Kit (Invitrogen). The PEDV RNA were detected using TaqMan real-time RT-qPCR with the following primers: sense, 5′-GAATTCCCAAGGGCGAAAAT-3′; antisense, 5′-TTTTCGACAAATTCCGCATCT-3′. A probe targeting the PEDV N gene (5′-FAM-CGTAGCAGGCTTGCTTCGGACCCA-BHQ-3′) was also employed. The thermal cycling parameters were as follows: 95°C for 20 s followed by 40 cycles at 95°C for 3 s and 60°C for 30 s.

### 2.8 Neutralization antibodies

Neutralization assays were conducted following a previously described method, with slight modifications (Song et al., 2024). Serum samples were heat-inactivated at 56°C for 30 minutes and serially diluted in twofold increments. The diluted samples were then mixed with PEDV hddz strain (200 TCID_50_) and incubated at 37°C for one hour. Subsequently, 0.1 mL of each mixture was added to a Vero cell monolayer in a 96-well tissue culture plate, pre-washed with DMEM. After 1.5 h of adsorption at 37°C, the inoculum was removed, and the cells were washed twice with PBS. A maintenance medium containing trypsin (7 µg/mL) was then added to each well, and the plate was incubated at 37°C for 48 h. The cells were monitored daily for cytopathic effects (CPE). Neutralizing antibody titers were determined as the highest serum dilution that protected more than 50% of the cells from CPE.

### 2.9 Histopathology and immunohistochemistry

The fixed intestinal tissues were dehydrated, embedded in paraffin, sectioned, mounted on slides, and stained with hematoxylin and eosin. The slides were examined using conventional light microscopy. To detect PEDV-specific antigens, selected formalin-fixed, paraffin-embedded sections were treated with PEDV-N-specific monoclonal antibodies (Zoonogen, China), followed by HRP-conjugated goat anti-mouse IgG secondary antibodies (Beyotime, China).

### 2.10 Statistical analysis

The graphs and statistical analyses in this study were performed using GraphPad Prism 8 (v8.0.2). Results are expressed as the standard error (SE) based on a minimum of three independent experiments.

## 3 Results

### 3.1 Expression and purification of PEDV S protein

We engineered a PEDV S protein expression system by fusing it with a T4 foldon sequence to enhance trimerization, and successfully expressed the protein in CHO K1 cells. The expression and purification of the PEDV S protein were confirmed through SDS-PAGE and Western blot analyses. SDS-PAGE revealed a single prominent band at the expected molecular weight (~200 kDa) (**Figure 1A**), which suggests that the S protein may contain glycosylation. The Western blot confirmed the identity of this band, demonstrating that the purified S protein retained its antigenic properties (**Figure 1B**). The SEC-HPLC analysis verified that the S protein predominantly forms a trimer. The chromatogram displayed a single dominant peak (nearing 650 kDa) corresponding to the molecular weight of the trimeric S protein. (**Figure 1C**). The TEM also confirmed the trimeric structure of the purified S protein (**Figure 1D**).

### 3.2 S protein subunit vaccine induces potent humoral immune responses in piglets

**Figure 2:**
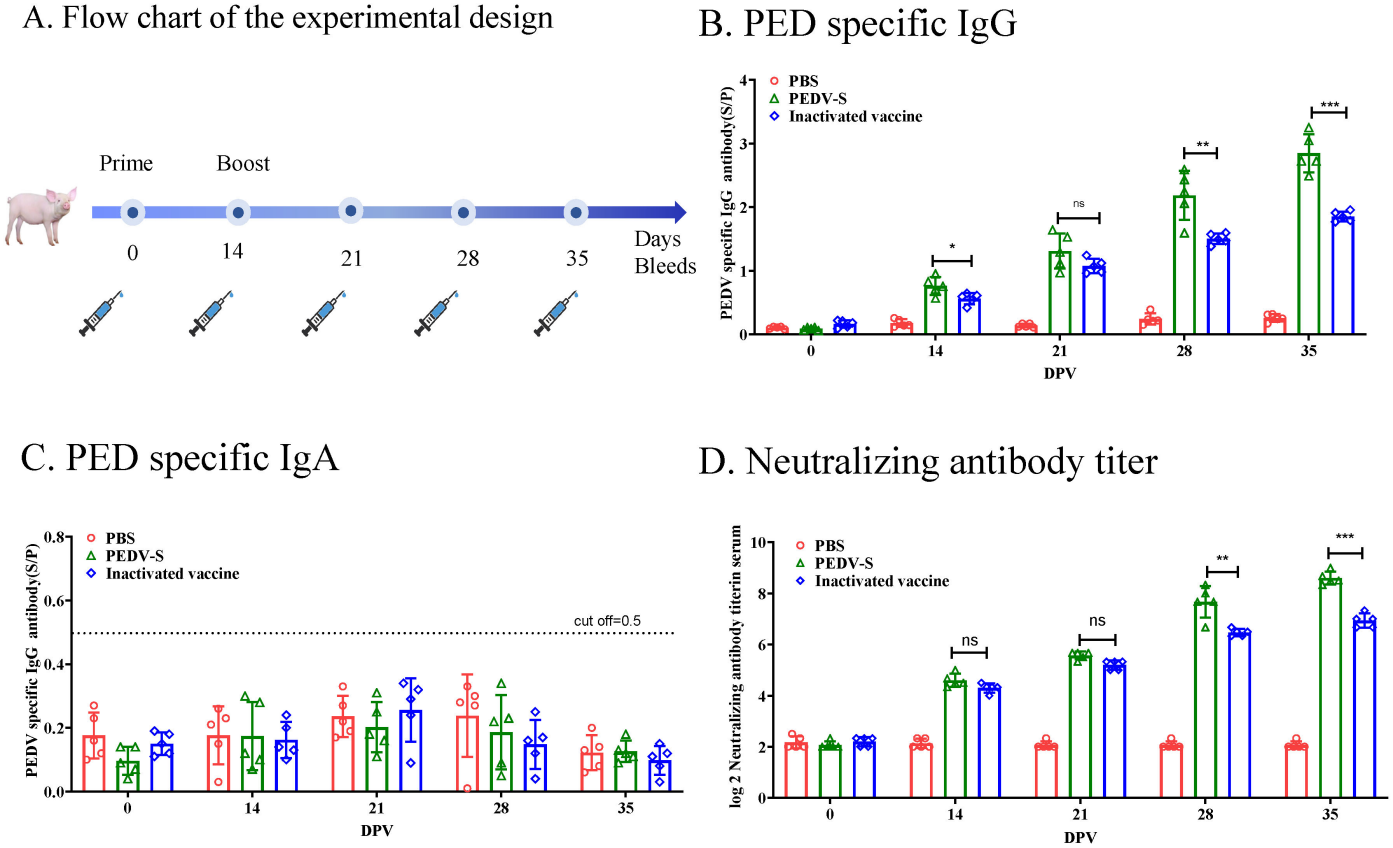
Humoral Immune Responses to the S Protein in Piglets. **(A)** Flow chart of the experimental design. Solid lines indicate the weeks of immunization, while syringes represent the weeks of sample collection. **(B)** PEDV-specific IgG antibodies in serum were measured using ELISA. **(C)** PEDV-specific IgA antibodies in serum were measured using ELISA. **(D)** Neutralizing antibody (NAb) titers were measured in serum samples through neutralization assays. Data are presented as the mean ± S.D. for five piglets per group. *p< 0.05, **p< 0.01, ***p< 0.001 indicate statistical significance.

To investigate the immunogenicity of the S protein subunit vaccine, piglets were immunized with subunit vaccines or inactivated PEDV vaccine formulated with ISA 201 VG adjuvant. Serum samples were collected at different time points post-immunization to evaluate the antibody responses using enzyme-linked immunosorbent assay (ELISA) or virus neutralization tests (**Figure 2A**). As showed in **Figure 2B**, the PED specific IgG antibody response to the PEDV S protein subunit vaccine turned positive 14 days after the initial immunization. Three weeks following the booster immunization, the IgG antibodies of the pigs immunized with S protein subunit vaccine were significantly higher levels than those observed in pigs immunized with the whole-virus inactivated vaccine. Interestingly, despite robust IgG responses, serum PEDV-specific IgA antibodies remained undetectable in both the subunit and inactivated vaccine groups, indicating a lack of mucosal immunity induction through this vaccination route (**Figure 2C**). In addition to the IgG response, neutralizing antibody titers were measured. Three weeks after the second immunization, the neutralizing antibody titer in the S protein subunit vaccine group reached 1:256 (**Figure 2D**), which was significantly higher than that induced by the whole-virus inactivated vaccine. These findings suggest that the S protein subunit vaccine elicits a stronger neutralizing antibody response.

### 3.3 S protein subunit vaccine induces potent humoral immune responses in sows

**Figure 3:**
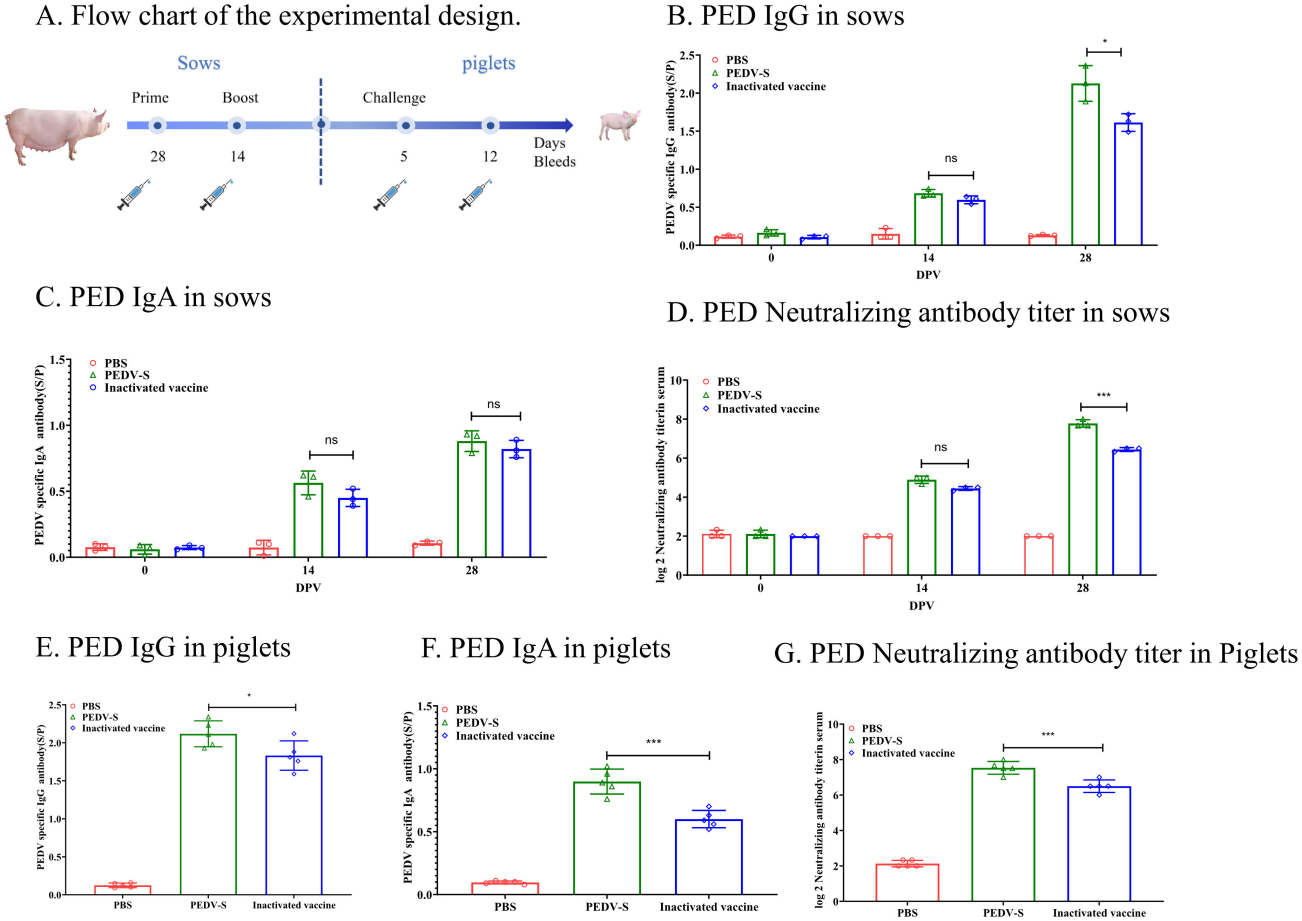
Humoral Immune Responses to the S Protein in Sows. **(A)** The flow chart illustrates the experimental design. Solid lines represent immunization weeks, while syringe icons denote the weeks of sample collection. **(B)** PEDV-specific IgG antibody levels in sow serum were quantified using ELISA. **(C)** PEDV-specific IgA antibody levels in sow serum were assessed using ELISA. **(D)** Neutralizing antibody titers in sow serum were determined through neutralization assays. **(E)** PEDV-specific IgG levels in piglet serum were measured after 5 days of suckling using ELISA. **(F)** PEDV-specific IgA levels in piglet serum were measured after 5 days of suckling using ELISA. **(G)** Neutralizing antibody (NAb) titers in piglet serum were measured after 5 days of suckling through neutralization assays.Data are presented as mean ± S.D. for three sows or five piglets per group, respectively. *p< 0.05, ***p< 0.001 indicate statistical significance.

To evaluate the protective efficacy of the PEDV S protein subunit vaccine, the sows immunized with the S protein unit vaccine or inactivated vaccine, and the PEDV IgG or IgA antibodies in the serum was detected by ELISA (**Figure 3A**). As showed in **Figure 3B**, The PEDV IgG antibodies in sow serum became positive at 14 DPI, and the PED IgG levels significantly increased by 14 days post-second immunization. Additionally, by 28 days post-immunization, the PED IgG antibody levels induced by the S protein subunit vaccine were significantly higher than those in the inactivated vaccine group. Although IgA antibodies became positive following vaccination, the overall IgA antibody levels remained low (S/P < 1.0) (**Figure 3C**). Importantly, 14 days after the second immunization, the average neutralizing antibody titer in the subunit vaccine group reached 1:256, which was significantly higher than the 1:64 observed in the inactivated vaccine group (**Figure 3D**). These results indicate that the S protein subunit vaccine elicits a more robust humoral immune response in sows compared to the inactivated vaccine. After 5 days of suckling, piglets born to sows immunized with the S protein subunit vaccine maintained higher levels of PEDV-specific IgG, IgA and neutralizing antibodies in their serum, suggesting effective transfer of maternal immunity (**Figures 3E–G**).

### 3.4 S protein-based subunit vaccine provides significant protection against P EDV dndz challenge in piglets

**Figure 4:**
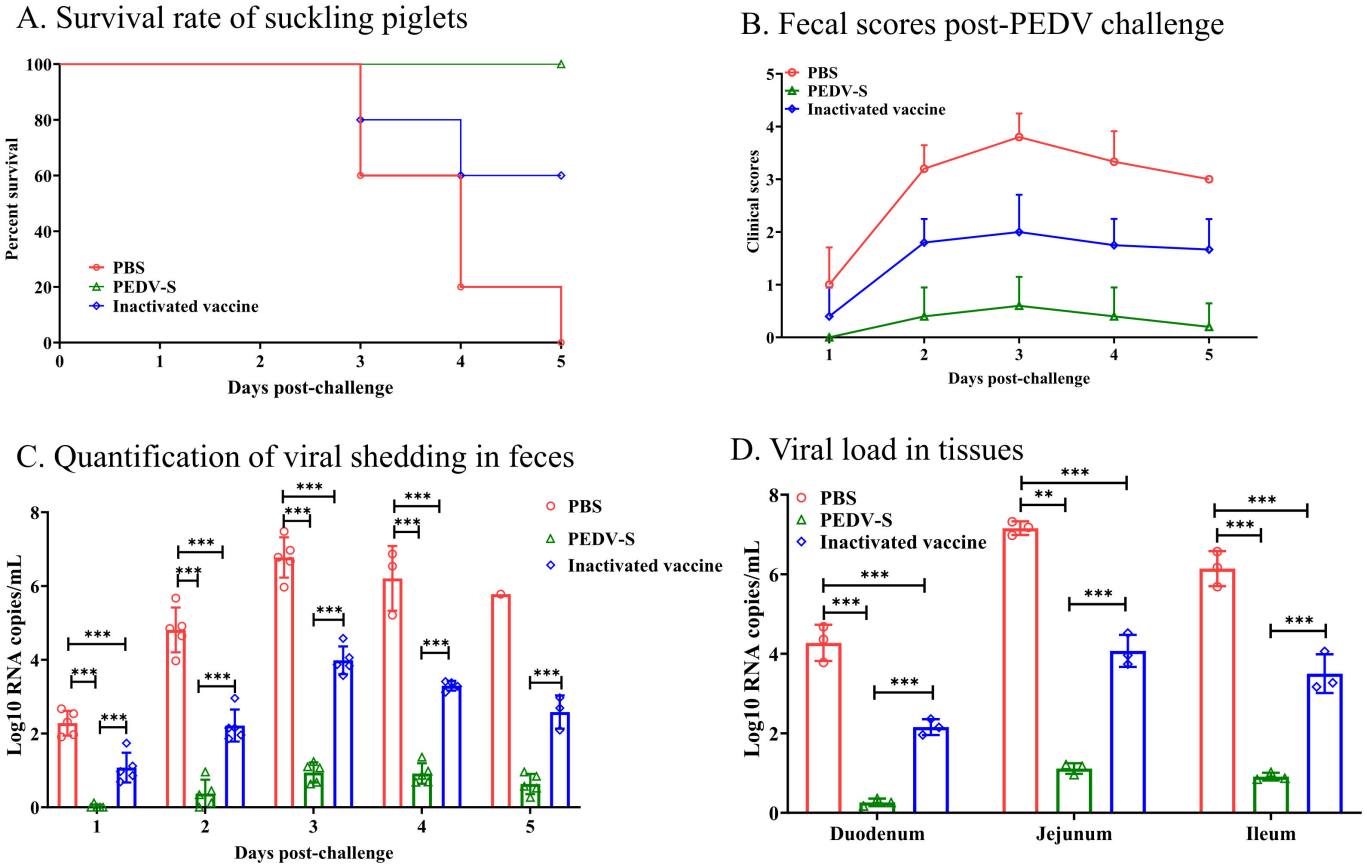
Protective efficacy of suckling piglets from sows vaccinated with the S protein vaccine. **(A)** Survival rate of suckling piglets post-PEDV challenge. **(B)** Fecal scores post-PEDV challenge. **(C)** Quantification of viral shedding in feces measured daily by qRT-PCR. **(D)** Viral load in the duodenum, jejunum, and ileum at 3 days post-infection (DPI). **p < 0.01, ***p< 0.001 indicate statistical significance.

Five piglets from each group were challenged with PEDV, and clinical symptoms and survival rates were monitored post-challenge. Additionally, three piglets from each group were challenged and necropsied on day 3 post-challenge to observe gross and microscopic lesions in the intestines. As shown in **Figure 4A**, by day 3 post-challenge, piglets in the control and inactivated vaccine groups experienced mortality, while all piglets in the S protein subunit vaccine group survived. Diarrhea onset was observed in the control group at 24 hpi, progressing to severe watery diarrhea by 48 h (**Figure 4B**). In the inactivated vaccine group, severe diarrhea occurred by 48 h post-challenge, whereas piglets in the S protein subunit vaccine group exhibited only mild diarrhea symptoms.

Rectal swabs were collected daily to assess viral load (**Figure 4B**). As shown in **Figure 4C**, the control group exhibited high viral shedding at 48 h post-challenge, peaking by day 3. In contrast, the S protein subunit vaccine group had significantly lower viral shedding, while the inactivated vaccine group showed intermediate shedding levels, lower than the control group but higher than the S protein subunit vaccine group. On day 3 post-challenge, intestinal tissues were collected for viral load analysis, which revealed that the control group had the highest viral load in the intestines. Although the inactivated vaccine group showed a reduction in viral load compared to the control group, it remained significantly higher than that of the S protein subunit vaccine group (**Figure 4D**).

These results indicate that immunizing sows with the PEDV S protein subunit vaccine provides enhanced passive immunity to their piglets through colostrum and milk, offering strong protection against a virulent PEDV challenge.

### 3.5 Gross pathology, histopathology, and immunohistochemistry

**Figure 5:**
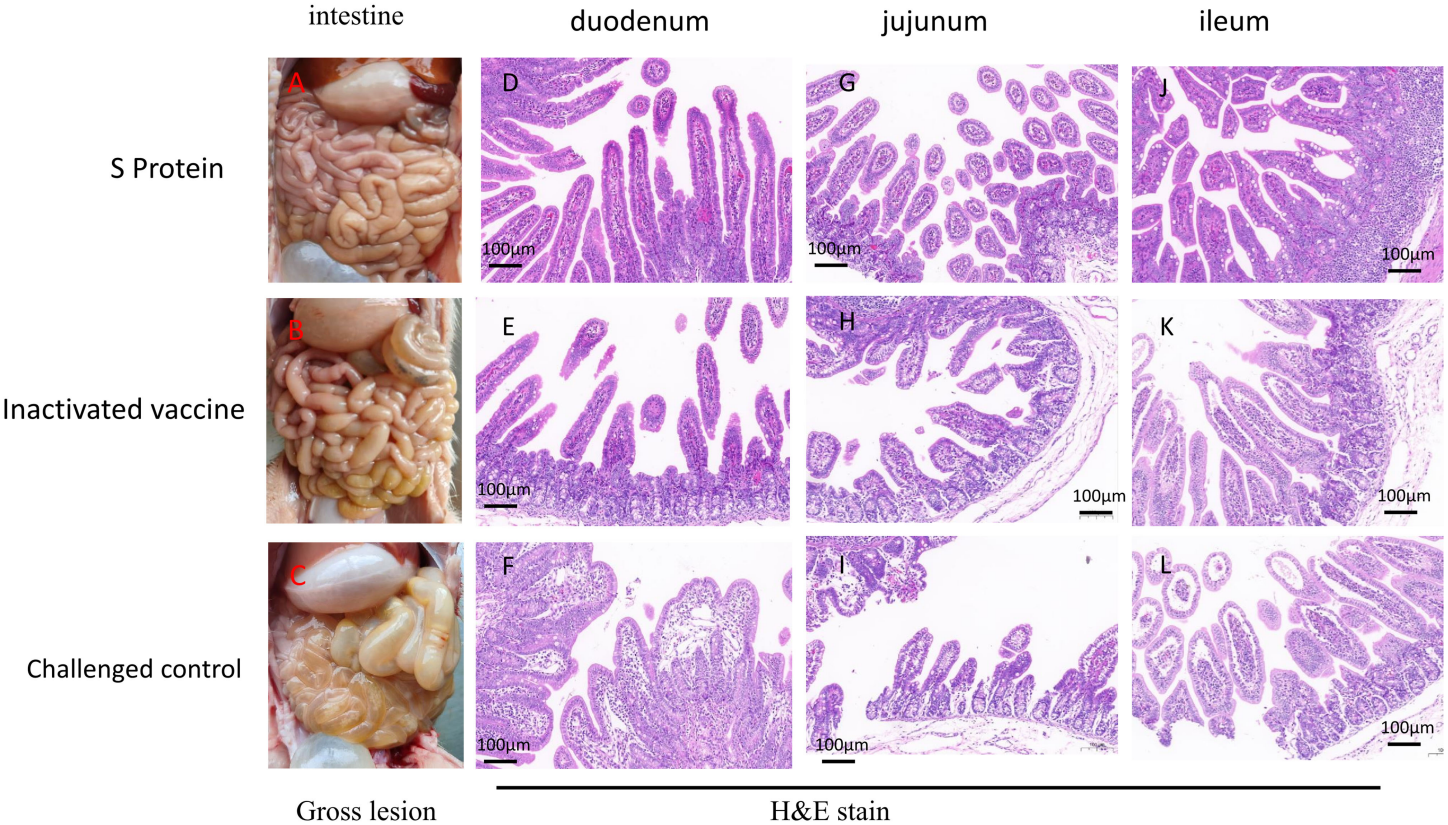
Gross and histological lesions in intestinal sections of piglets euthanized three days post-challenge. Representative intestinal sections were obtained through post-mortem examination and stained with H&E. Gross lesions of the piglets in the S protein group **(A)**, inactivated vaccine group **(B)** or challenged control group **(C)**. Histopathological Sections of the duodenum **(D)**, jejunum **(G)**, and ileum **(J)** in the S protein Group. Histopathological Sections of the duodenum **(E)**, jejunum **(H)**, and ileum **(K)** in the inactivated vaccine group. Histopathological Sections of the duodenum **(F)**, jejunum **(L)**, and ileum **(I)** in the challenged control group. Scale bar: 100 µm.

Gross pathology and histopathology tests were conducted at 3 DPC. Upon necropsy, piglets from the S protein subunit vaccine group showed no observable intestinal lesions post-challenge, indicating effective protection against PEDV-induced intestinal damage (**Figure 5A**). In contrast, piglets from the inactivated vaccine group exhibited mild pathological changes, including slight thinning of the small intestine, increased transparency, and the presence of watery intestinal contents (**Figures 5B, E, H, K**). The control group, which did not receive any vaccine, displayed severe intestinal pathology, characterized by pronounced thinning and transparency of the small intestine, along with gas accumulation and watery contents (**Figures 5C, F, I, L**), indicative of severe PEDV infection.

In the challenge control group, microscopic examination of the duodenum revealed villous underdevelopment and moderate dilation of the central lacteals (**Figure 5F**). In the jejunum, there was villous underdevelopment, necrosis, and shedding of epithelial cells, along with mild dilation of the central lacteals and underdevelopment of the submucosal glands (**Figure 5I**). The ileum also showed villous underdevelopment and edema of the lamina propria (**Figure 5L**). These findings indicate significant structural damage in the intestines caused by the PEDV infection. In contrast, piglets from the S protein subunit vaccine group exhibited well-preserved intestinal structures in the duodenum, jejunum, and ileum, with intact villi and no signs of inflammation (**Figures 5D, G, J**). In the inactivated vaccine group, the duodenum, jejunum, and ileum displayed signs of underdevelopment in the villi and submucosal glands, with edema observed in the lamina propria of the jejunum and ileum. However, no inflammatory reactions were noted, suggesting partial protection but still noticeable intestinal damage compared to the subunit vaccine group (**Figures 5E, H, K**).

**Figure 6:**
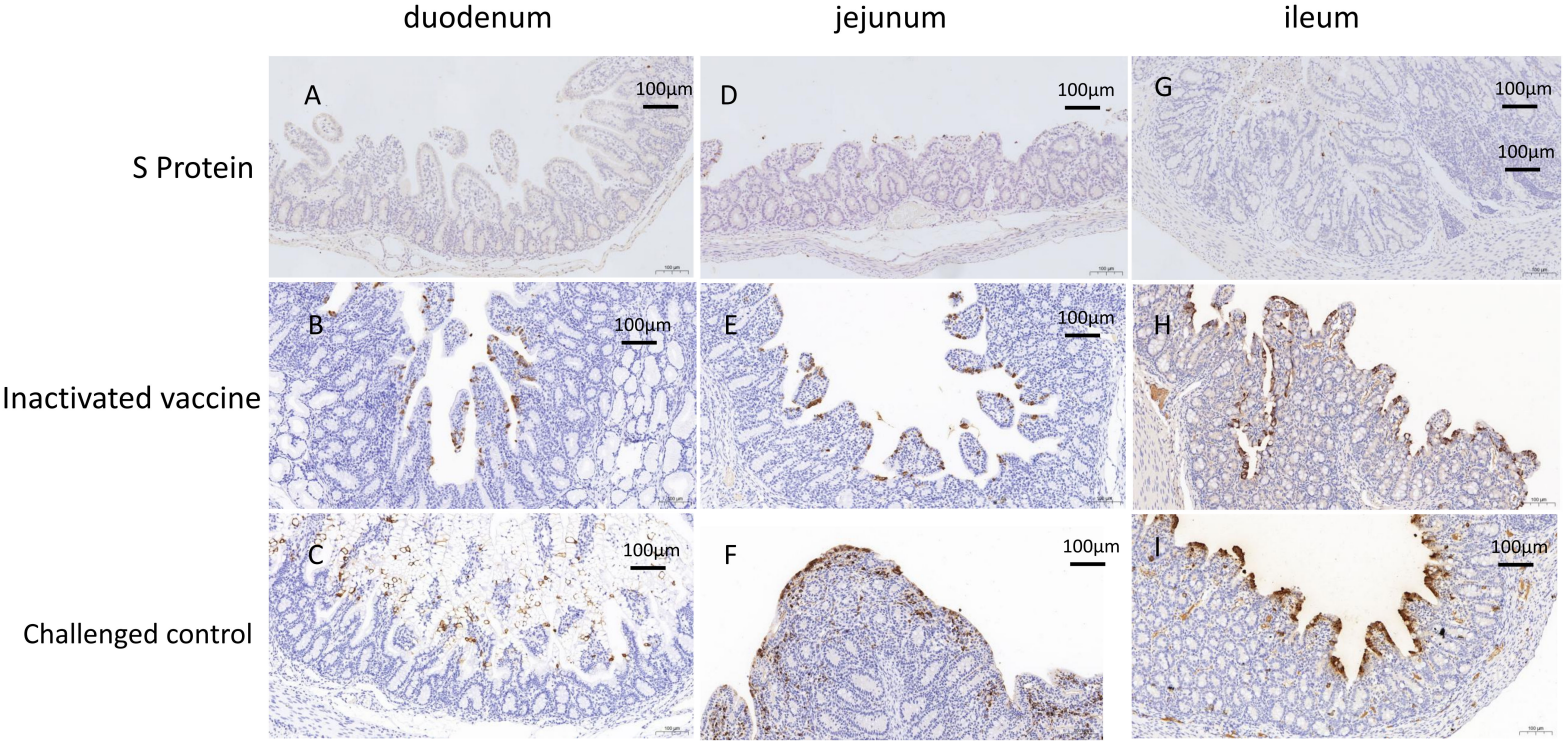
Immunohistochemistry (IHC) analysis of the piglets euthanized three days post-challenge. IHC analysis of the duodenum **(A)**, jejunum **(D)**, and ileum **(G)** in the S protein Group. IHC analysis of the duodenum **(B)**, jejunum **(E)**, and ileum **(H)** in the inactivated vaccine group. IHC analysis of the duodenum **(C)**, jejunum **(F)**, and ileum **(I)** in the challenged control group. Scale bar: 100 µm.

Immunohistochemistry results supported these findings (**Figure 6**). In the challenge control group, extensive positive signals were detected in the epithelial cells of the duodenal, jejunal, and ileal villi, as well as in the necrotic epithelial cells. Scattered positive signals were also found in the lymphoid tissues of the lamina propria and submucosa, indicating widespread viral presence (**Figures 6C, F, I**). In the S protein subunit vaccine group, only minimal positive signals were observed in the villi and lymphoid tissues (**Figures 6A, D, G**), suggesting a low level of viral presence. The inactivated vaccine group showed a higher number of positive signals in the epithelial cells of the villi and necrotic cells compared to the subunit vaccine group (**Figures 6B, E, H**), indicating a greater viral load but still less than the challenge control group.

These findings highlight the superior protection provided by the S protein subunit vaccine in preserving intestinal structure and minimizing viral presence, compared to the partial protection observed with the inactivated vaccine.

## 4 Discussion

Since its re-emergence from 2010, PEDV has caused substantial economic losses in the global swine industry due to the high mortality in young piglets and reduced productivity in affected farms (Zhang et al., 2023). Vaccination with inactivated or live-attenuated vaccines are the major control measures to prevent the disease. Although commercial vaccines have been used in pig farms in China, the epidemic of PEDV cause huge economic losses in China (Wang et al., 2024; Zhang et al., 2023). Wild-type PEDV feed-back measure can induced mucosal immunity in sows and the IgA from the milk can protect piglets against PEDV attacks (Hu et al., 2024; Langel et al., 2016). However, this method leads to the continuous spread of the virus in the field, making it very difficult for pig farms to achieve virus elimination. Therefore, the development of safer and more effective vaccines is critical for the sustainable control of PEDV outbreaks.

The spike glycoprotein of coronaviruses is the major target for vaccine development (Aram et al., 2024; Wang et al., 2024). The coronavirus Spike protein is a glycosylated trimeric protein. Therefore, subunit vaccines for coronaviruses like SARV-CoV often use mammalian cells such as CHO cells for expression in which retains a natural conformation similar to the virus, leading to improved immunogenicity (Ahuja et al., 2024; Jitender et al., 2024). Previous studies have shown that PEDV mRNA vaccines and S protein vaccines can induce the production of high levels of neutralizing antibodies, providing piglets with good passive immunity protection and resistance against severe PEDV attacks (Song et al., 2024; Zhao et al., 2024).

In this study, we demonstrate the superior immunogenicity and protective efficacy of the PEDV S protein subunit vaccine compared to the traditional inactivated vaccine. The subunit vaccine induced significantly higher levels of IgG and neutralizing antibodies in both sows and piglets, offering better protection against PEDV. This is evidenced by reduced viral shedding, milder clinical symptoms, and fewer intestinal lesions in piglets born to immunized sows. At birth, serum PEDV-specific IgG and IgA in piglets were both negative, but after suckling, the piglets’ serum IgG antibodies became positive, with a high level of neutralizing antibodies, indicating that maternal antibodies can be transferred to piglets through colostrum. These findings suggest that the S protein subunit vaccine facilitates stronger humoral immunity, resulting in a more effective passive transfer of maternal antibodies to newborn piglets through colostrum and milk. The PDCoV S subunit vaccine can also induce high levels of neutralizing antibodies, which are transferred from sows to piglets, providing a good protective effect (Li et al., 2023).

Despite these advantages, challenges remain for widespread implementation of subunit vaccines. Production costs can be higher than traditional vaccines, and optimization of adjuvants and delivery methods is necessary to maximize their efficacy. Nonetheless, the results from this study underscore the potential of the PEDV S protein subunit vaccine as a safer and more effective alternative for controlling PEDV outbreaks.

In conclusion, while current PEDV vaccines provide a degree of protection, the development of more effective vaccines, such as the S protein subunit vaccine, is essential to achieving better disease control. Future research should focus on refining vaccine formulations, assessing long-term immunity, and exploring large-scale field applications to further reduce the economic impact of PEDV on the swine industry.

## Data Availability

The original contributions presented in the study are included in the article/Supplementary Material. Further inquiries can be directed to the corresponding author.
